# A new era for functional labeling of neurons: activity-dependent promoters have come of age

**DOI:** 10.3389/fncir.2014.00037

**Published:** 2014-04-23

**Authors:** Takashi Kawashima, Hiroyuki Okuno, Haruhiko Bito

**Affiliations:** ^1^Department of Neurochemistry, Graduate School of Medicine, The University of TokyoTokyo, Japan; ^2^Core Research for Evolutionary Science and Technology, Japan Science and Technology AgencySaitama, Japan

**Keywords:** *Arc*, neuronal ensemble, E-SARE, live imaging, activity-dependent gene expression, c-*fos*

## Abstract

Genetic labeling of neurons with a specific response feature is an emerging technology for precise dissection of brain circuits that are functionally heterogeneous at the single-cell level. While immediate early gene mapping has been widely used for decades to identify brain regions which are activated by external stimuli, recent characterization of the promoter and enhancer elements responsible for neuronal activity-dependent transcription have opened new avenues for live imaging of active neurons. Indeed, these advancements provided the basis for a growing repertoire of novel experiments to address the role of active neuronal networks in cognitive behaviors. In this review, we summarize the current literature on the usage and development of activity-dependent promoters and discuss the future directions of this expanding new field.

## INTRODUCTION

The central nervous systems of animals, including insects, vertebrates, and primates, are controlled by coordinated action of multiple brain regions. Each brain region consists of thousands of homologous neurons composed of multiple anatomical types. Interestingly, functional neuronal subsets that share similar response features or are coactivated during one behavioral task are usually sparsely scattered within homologous neural populations. For example, in the visual cortex of rodents, neurons of different orientation preferences are intermingled together at the single-cell level (Ohki et al., 2005). Thus, for precise understanding of circuit dynamics underlying cognitive brain function at the cellular level, it is of primary importance to selectively label these functionally defined subsets of neurons and analyze their nature in detail.

One approach towards this goal is to use genomic sequences, e.g., promoters and enhancers, that control the expression of neuronal immediate early genes (IEGs) which are rapidly induced by neuronal activity (Smeyne et al., 1992; Barth, 2007; Inoue et al., 2010; Okuno, 2011). The mechanisms underlying such gene induction events have been characterized in detail in the past. When a neuron receives large amounts of synaptic inputs, calcium ions rapidly flow into neurons through synaptic N-methyl-D-aspartic acid (NMDA) receptors or voltage-gated calcium channels (VGCCs). This turns on several calcium-dependent kinase cascades, which then activate a set of transcription factors and trigger rapid induction of target genes (Bito et al., 1997; Flavell and Greenberg, 2008). In combination with a variety of reporter proteins that can be driven to label neurons, IEG promoters have thus been exploited to map neuronal activation patterns associated with a specific animal sensation or behavior at a cellular resolution.

Among neuronal IEGs, promoters for c-*fos* (Schilling et al., 1991) and *Arc/Arg*3.1 (Waltereit et al., 2001; Kawashima et al., 2009; Pintchovski et al., 2009) were well characterized and widely used as a tool to facilitate neuronal activity mapping *in situ* on histological sections. The availability of novel genetic methods to artificially manipulate neural activity further provided an opportunity to expand the application of activity-dependent promoters, to address new questions in anatomical, electrophysiological, and cognitive research. The promoter of c-*fos* was successfully employed for investigating the formation of memory engram in rodents (Garner et al., 2012; Liu et al., 2012; Ramirez et al., 2013). Furthermore, the promoter of *Arc/Arg*3.1 was successfully employed for live imaging of cortical activation both at the cellular scale (Wang et al., 2006; Kawashima et al., 2013) and at the whole-cortex scale (Eguchi and Yamaguchi, 2009; Izumi et al., 2011). Recently, a novel activity-dependent promoter E-SARE (enhanced synaptic activity-responsive element) was engineered, which enabled an activity-based, long-distance axonal tracing in living animals (Kawashima et al., 2013). In this review, we summarize the latest advances and discuss future directions of this expanding new field.

## THE RISE OF BRAIN ACTIVITY MAPPING BASED ON IEG EXPRESSION

During the 1970s, several important strategies were developed to selectively label activated brain regions. The first visualization was achieved by the autoradiography of a metabolic marker 2-deoxy-D-glucose (2-DG; Sokoloff et al., 1977). 2-DG was incorporated into tissues with ongoing high energy consumption and, since it could not undergo glycolysis, remained in the incorporated tissue. In the brain, 2-DG was incorporated into active brain regions, serving as a marker for neuronal activation. A less invasive, blood oxygenation level dependent (BOLD) contrast-based method further enabled functional magnetic resonance imaging of live brain activity (Ogawa et al., 1990). However, these whole-brain tissue imaging showed only limited spatial resolution and did not confer cellular resolution.

The first activity mapping with cellular resolution was achieved by immunostaining of inducible gene expression. Preceding studies showed that the proto-oncogene c-*fos* in cultured cells could be rapidly induced by application of growth factors (Greenberg and Ziff, 1984; Müller et al., 1984; Curran and Morgan, 1985). Taking advantage of this, neurons in the brain that were activated by electrical seizure, tactile stimulation, and water starvation were visualized by immunostaining of c-Fos (Morgan et al., 1987; Sagar et al., 1988). Largely based on these pioneering studies, monitoring of up-regulated expression of IEGs, such as c-*fos* or *zif*268/*egr*-1 (Saffen et al., 1988), and also of activated transcription factors, such as phosphorylated cAMP response element-binding protein (CREB) (Ginty et al., 1993; Bito et al., 1996; Deisseroth et al., 1996), either using immunostaining or *in situ* hybridization, has become accepted as a widely applicable method to map activated circuits at cellular scale.

Attempts to identify activity-induced genes in the hippocampus led to the cloning of a new IEG, *Arc* (aka *Arg*3.1; Link et al., 1995; Lyford et al., 1995). Subsequently, the unique transport kinetics of *Arc* mRNAs from nucleus to cytoplasm was exploited to build a catFISH (cellular compartment analysis of temporal activity by fluorescent *in situ* hybridization) assay: by examining the differential localization of *Arc* mRNA, distinct neuronal ensembles that were activated in two different environments at a 20 min interval could be clearly discriminated (Guzowski et al., 1999). Detailed spatiotemporal analyses of *Arc* expression further indicated a strong correlation between *Arc* induction with cognitive behavior in normal as well as aged animals (Small et al., 2004; Ramírez-Amaya et al., 2005; Hartzell et al., 2013).

However, the signal-to-noise ratio of gene expression mapping heavily depended on the labeling protocol and the quality of antibodies or hybridization probes, which sometimes caused high variability/low reliability of the mapping results. To address this issue, the promoter of the c-*fos* gene was isolated and fused with an expression cassette of a reporter protein, beta-galactosidase (LacZ; Schilling et al., 1991). This led to a generation of *fos-lacZ* transgenic mice and enabled the activity mapping at a cellular scale with higher signal-to-noise ratio based on β-gal staining (Smeyne et al., 1992; Robertson et al., 1995). These studies also demonstrated the utility of IEG promoters as a versatile tool to label a functionally defined subset of neurons within a heterogeneous neural population.

## BRAIN ACTIVITY MAPPING BASED ON ACTIVITY-DEPENDENT PROMOTERS

Following the generation of the first line of *fos-lacZ* mice, several other transgenic mice that expressed *lacZ* downstream of activity-dependent promoters were generated such as cAMP response element (CRE)-*lacZ* (Impey et al., 1996), *egr*-1-*lacZ* (Tsai et al., 2000) and *fos-tau-lacZ* (Wilson et al., 2002) mice. These *lacZ* transgenic mice enabled histological visualization of active circuits during seizure (Smeyne et al., 1992), development during critical period (Pham et al., 1999) and water deprivation (Wilson et al., 2002). The reliance on LacZ as a reporter, however, required fixation and staining of tissue sections, and this method was therefore not suited for anatomical and physiological investigation of live animals. Furthermore, the relative stability of the LacZ reporter protein caused a high signal background, thus hampering a clean mapping of authentic neural activity.

With the advent and improvement of fluorescent proteins, the destabilized green fluorescent protein (GFP) has become the preferred choice as a reporter of activity-dependent promoters in transgenic mice and virus vectors (Barth et al., 2004; Wang et al., 2006; Eguchi and Yamaguchi, 2009; Grinevich et al., 2009; Kawashima et al., 2009; Okuno et al., 2012). As GFP fluorescence enabled high S/N observation of live neurons, these mice and virus vectors widely expanded the scope of gene expression mapping based on activity-dependent promoters. Two-photon fluorescence microscopy visualized orientation-specific neuronal activation in living animals at single-cell resolution (Wang et al., 2006; Kawashima et al., 2013). Fluorescent live imaging of whole cortical areas revealed brain regions that were visually activated (Eguchi and Yamaguchi, 2009; Grinevich et al., 2009). The use of a bioluminescent protein, *firefly* luciferase, also enabled visualization of plastic changes in sensory cortices after sensory deprivation (Wada et al., 2010; Izumi et al., 2011).

One drawback of activity mapping based on histological methods is the time-consuming and labor-intensive processing and analysis of histological sections. However, a recent technological advancement, a serial two-photon tomography (STP; Ragan et al., 2012), holds great promise for overcoming this bottleneck. This technique allows automated acquisition of fluorescent images of a histological block surface, which is sequentially cut by an automated microtome. This dramatically reduces the burden of manually preparing serial histological sections and enables serial acquisition of fluorescent images of the entire mouse brain within a couple of days. When appropriately combined with transgenic mice expressing fluorescent reporters downstream of activity-dependent promoters (Osten and Margrie, 2013), this technique provides whole-brain datasets of cellular level activation under various behavioral conditions (Vousden et al., in press).

## NEURONAL CELL TYPES LABELED BY THE ACTIVITY-DEPENDENT GENE PROMOTERS

Accumulating histological evidences suggest that activity-dependent genes are differentially regulated in different cell types in distinct brain areas. In the neocortex and the hippocampus, IEGs are mostly up-regulated in excitatory pyramidal neurons (Chaudhuri et al., 1995; Filipkowski, 2000), although inhibitory neurons can also express some IEGs, such as c-*fos* and *Arc*, after strong stimulation (Staiger et al., 2002; Vazdarjanova et al., 2006). In contrast, inhibitory granule cells, rather than excitatory mitral cells, mainly express c-*fos* and *Arc* in the olfactory bulb (Guenthner et al., 2013). In the striatum, *Arc* and *egr-*1, but not c-*fos*, are strongly expressed by GABAergic medium spiny neurons (Moratalla et al., 1993; Vazdarjanova et al., 2006; Guenthner et al., 2013). In contrast, thalamic areas tend to express c-*fos* rather than *Arc* or *egr*-1 (Steiner and Gerfen, 1994; Link et al., 1995; Lyford et al., 1995). In the cerebellum, c-*fos* is more expressed than *Arc* in excitatory granule cells (Guenthner et al., 2013), while both c-*fos* and *Arc* can be expressed in cerebellar Purkinje cells (Tian and Bishop, 2002; Smith-Hicks et al., 2010; Mikuni et al., 2013).

Such a variety in cell-type and regional specificity of activity-regulated IEG expression is thought arise from a combination of distinct transcriptional regulation and cellular calcium kinetics. Reporter expression from isolated promoter elements also appears to recapitulate the cell-type or regional preference of the original genes (Eguchi and Yamaguchi, 2009; Yassin et al., 2010; Kawashima et al., 2013). This suggests that it is of primary importance to select the type of activity-dependent promoters depending on the target cell type and brain region in which one wishes to achieve significant activity-dependent reporter expression. Also, to the best of our knowledge, there are as yet no IEGs that are expressed exclusively in a particular cell type, such as GABAergic or dopaminergic. It would thus seem a worthy challenge to seek and characterize novel IEGs and design new synthetic activity-dependent promoters that possess restricted cell-type preferences.

## MECHANISMS UNDERLYING ACTIVATION OF ACTIVITY-DEPENDENT GENE PROMOTERS

The mechanisms underlying activation of IEG promoters have been the subject of detailed studies for decades. These core regulatory mechanisms may be largely divided into three steps: influx of calcium ions triggered by synaptic inputs and neural firing, activation of calcium-dependent kinase cascades, and activation of transcription factors by kinases (Bito et al., 1997; Flavell and Greenberg, 2008). When a neuron receives intense synaptic inputs, calcium ions flow into the cytoplasm through NMDA-type glutamate receptors (NMDARs) present at activated synapses as well as through VGCCs that open upon neuronal firing. This in turn stimulates the activation of several calcium-dependent kinase cascades, that comprise Ca^2^^+^/calmodulin-dependent protein kinases (CaMKs; Bito et al., 1996; Fujii et al., 2013) and mitogen-activated protein kinases (MAPKs; Dolmetsch et al., 2001; Zhai et al., 2013). Finally, activation of these kinases cascades leads to site-specific modulation of activity-dependent transcription factors, such as CREB (Bito et al., 1996), myocyte enhancer factor-2 (MEF2; Mao et al., 1999), and serum-responsive factor (SRF; Norman et al., 1988), thereby turning on rapid transcription of downstream IEGs.

We recently discovered a SARE from the *Arc* promoter/enhancer region (Kawashima et al., 2009). Our studies on SARE revealed that three different transcription factors, CREB, MEF2, and SRF, cooperated within this ~100 bp locus to induce activity-dependent transcription that was substantially more potent than through the action of each individual factor alone. This finding demonstrated that activity-dependent promoters performed a supralinear integration of the output of several co-activated signaling pathways, in keeping with previous analyses on c-*fos* (Robertson et al., 1995) and *bdnf* promoters (West et al., 2001).

At the physiological level, activation of IEG promoters is thought to be an event critical for the conversion of short-term stimuli that transiently activate neurons (with a timescale of milliseconds to minutes) into a long-term neuronal plasticity that requires gene expression (with a timescale of days to years). Such an ability to re-scale information in the time axis seems to be one of the key features of IEG promoter activity, and this may be pivotal in producing persistent traces of neuronal activity. Thus, once a subset of neurons received external stimuli during plasticity induction, they may express a “plasticity-related reporter protein” for a certain period of time (from several hours to a couple of days). Consistently blockade of activity-dependent gene expression pathways led to strong impairment of late-phase LTP and long-term memory without affecting early phase LTP and short-term memory (Bourtchuladze et al., 1994; Abel et al., 1997; Kang et al., 2001; Kida et al., 2002).

## ENGINEERING A POTENT SYNTHETIC ACTIVITY-DEPENDENT PROMOTER

Although a large amount of effort has been spent in the past on the application of activity-dependent IEG promoters, very little effort was made to improve endogenous promoters, and create an experimentally optimized synthetic activity-dependent promoter. Notable exceptions were the use of synthetic binding sites for activity-regulated transcription factors (such as multiplexed CRE) to drive expression of several reporter proteins (Impey et al., 1996; Böer et al., 2007). In most of these cases, however, the expression levels of the reporters remained relatively weak, presumably because these artificial promoters did not adequately replicate the coordinated action of multiple transcription factors as found in the context of endogenous IEG promoters (Kawashima et al., 2009).

**FIGURE 1:**
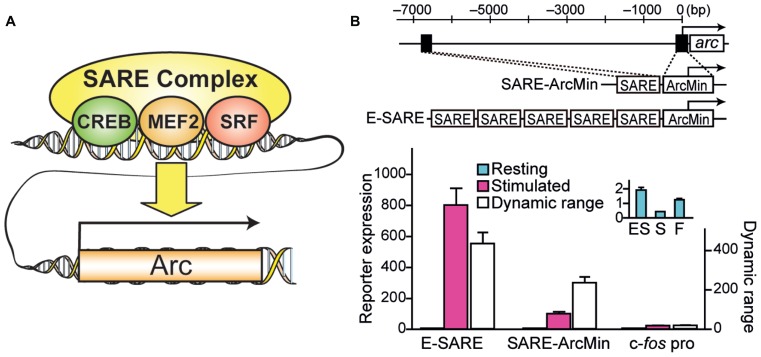
**Engineering of a synthetic E-SARE promoter. (A)** The structure of SARE enhancer element which regulates activity-dependent expression of *Arc/Arg3.1*. Three distinct transcription factors, CREB, MEF2, and SRF, cooperate to induce strong transcription. **(B)** Top, construction strategy of E-SARE promoter. The locations of SARE and TATA-containing ArcMin in the *Arc* promoter are represented by black boxes. E-SARE is constructed by connecting SARE elements in tandem. Bottom, comparison of E-SARE with SARE-ArcMin and c-*fos* promoters by luciferase reporter assay in cultured neurons under resting (blue) and stimulated (pink) conditions. Dynamic ranges (calculated based on the ratio between stimulated and resting conditions; white) are also shown. Inset, expanded values obtained under resting conditions. Adapted from Kawashima et al. (2013).

Recently, a novel engineered synthetic promoter, E-SARE, was successfully constructed based on the SARE enhancer element of the *Arc* promoter (Kawashima et al., 2009, 2013; **Figure 1**). The SARE element had a unique “modular” structure: it was short (~100 base pairs); it had cooperative binding sites for three activity-dependent transcription factors, CREB, MEF2, and SRF/TCF; and this element was sufficient to induce a strong transcription independently of the type of the downstream minimal promoters that contains TATA-box (Kawashima et al., 2009). To generate an enhanced promoter based on this modularity, multiple SARE elements were connected in tandem with an adequate linker length. We found combinations with a 5 tandem repeat to yield a sevenfold increase in the reporter expression level. The resulting synthetic promoter, E-SARE, had more than 20-fold higher expression level and 30-fold higher induction ratio than the c-*fos* promoter (Kawashima et al., 2013; **Figure 1**).

The discovery of SARE and the resulting rational synthesis of the E-SARE promoter was a significant breakthrough, not only because this expanded the palette of available activity-regulated promoters, but mainly because this greatly facilitated the introduction of viral vector strategies into the labeling of active neuronal ensemble. Because of their shortness (<1000 base pairs) and high reporter expression, SARE and E-SARE can be implemented into viral vector backbones such as lentivirus (LV) and adeno-associated virus (AAV), both of which have relatively limited genomic capacities, while allowing substantial flexibility in expression cassette design. As compared with developing transgenic rodents, viral vectors have the further advantage of requiring only a shorter time and much lower cost for production. Finally, it also opens a new avenue by potentially enabling an active ensemble mapping in larger mammalian species other than rodents, such as non-human primates, whose genomic engineering remains technically more challenging.

**Table 1** summarizes a list of previously published transgenic animals and viral vectors that took advantage of various activity-dependent promoters to drive a reporter protein in the brain. The downstream reporter proteins were limited to LacZ and EGFP until late 2000s. With the explosion of newly available genetic methods, the genetic resources based on activity-dependent promoters have much broadened in scope and in sophistication (**Table 1**).

**Table 1:** Reporter mouse lines and viruses based on activity-dependent promoters.

Gene	Type of promoter	Reporter protein	Resource type	Reference
**c-*fos***	Mouse 5′ regulatory region (600 base pairs)	cFos-LacZ fusion	Transgenic mouce	Smeyne et al. (1992)
		Tau-LacZ fusion		Wilson et al. (2002)
		cFos-EGFP fusion		Barth et al. (2004)
		*firefly* luciferase		Geusz et al. (1997)
		cFos-LacZ fusion	Transgenic rat	Kasof et al. (1995)
		cFos-EGFP fusion		Cifani et al. (2012)
		PSD95-Venus fusion		Knapska et al. (2012)
		tTA\d2EGFP	Double transgenic mouse	Reijmers et al. (2007)
	Knock-in	CreER^T2^	Knock-in mouse	Guenthner et al. (2013)
***Arc/Arg3.1***	Knock-in	d2EGFP	Knock-in mouse	Wang et al. (2006)
		CreER^T2^		Guenthner et al. (2013)
	Mouse 5′regulatory region (7k base pairs)	d2Venus	Transgenic mouse	Eguchi and Yamaguchi (2009), Mikuni et al. (2013)
		EGFP-Arc fusion		Okuno et al. (2012)
	mouse BAC clone	d4EGFP		Grinevich et al. (2009)
		*firefly* luciferase		Izumi et al. (2011)
	SARE enhancer	d2EGFP	Lentiviral vector	Kawashima et al. (2009)
	E-SARE promoter	d2EGFP	Adeno-associated virus	Kawashima et al. (2013)
		ER^T2^-Cre-ER^T2^		
**Egr-1**	Mouse 5′ regulatory region (1.5 K base pairs)	d2EGFP	Transgenic rat	Man et al. (2007)
**CREB binding sites**	6xCRE-RSV promoter	LacZ	Transgenic mouse	Impey et al. (1996)
	4xCRE-TK promoter	*firefly* Luciferase		Böer et al. (2007)

*Note that this is not an exclusive survey.*

## ELECTROPHYSIOLOGICAL CHARACTERIZATION OF NEURONS LABELED WITH ACTIVITY-DEPENDENT PROMOTERS

Despite our detailed understanding of the molecular pathways leading to activity-dependent gene expression, we understand much less about the input patterns of endogenous neuronal activities that trigger activity-dependent gene expression *in vivo.* Early studies demonstrated that *in vivo* high-frequency stimulation of medial perforant path axons from the entorhinal cortex triggered *Arc* mRNA expression in the dentate gyrus of the hippocampus (Link et al., 1995; Steward et al., 1998). *In vitro* experiments using cultured neurons further indicated that both LTP-inducing and LTD-inducing stimuli induces phosphorylation of an activity-dependent transcription factor CREB (Deisseroth et al., 1996). Consistently, either repeated high-frequency bursts (50Hz) or prolonged, low- frequency stimuli (5Hz) was shown to induce c-Fos expression (Bito et al., 1996).

**FIGURE 2:**
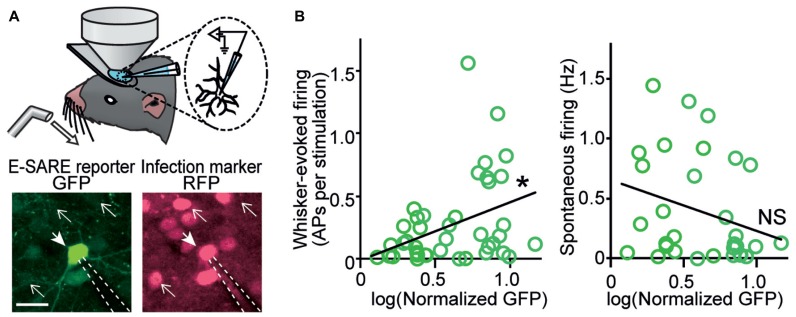
***In vivo* recording reveals a strong correlation between evoked neuronal activity and E-SARE expression. (A)** Top, experimental setup. Single-cell responses of E-SARE-driven GFP-positive and – negative neurons were recorded under the observation by two-photon microscopy. Bottom, representative images of a recorded neuron that is GFP reporter-positive (filled arrow). The dashed lines and line arrows indicate the recording electrode and GFP-negative neurons, respectively. Scale bar, 20 μm. **(B)** GFP brightness of recorded neurons correlates with their whisker-evoked activity (left) but not with spontaneous activity rate (right). Adapted from Kawashima et al. (2013).

However, electrophysiological characterization of individual neurons in which activity-dependent transcription has been turned on in living animals was achieved much later. Two studies based on single-cell recording guided by *in vivo* two-photon fluorescent microscopy provided critical insights into the type of neuronal activity which triggers activity-dependent gene expression. One study was based on FosGFP transgenic mice, in which a cFos-GFP fusion protein was expressed downstream of a c-*fos* promoter (Barth et al., 2004). This study showed that an increased spontaneous activity was correlated with the FosGFP expression in layer 2/3 pyramidal neurons of the somatosensory barrel cortex (Yassin et al., 2010). Because synaptic connectivity was enriched between FosGFP-positive neurons, the results were indicative of a functional sub-network of highly excitable neurons sparsely embedded in the barrel cortex (Yassin et al., 2010). Another study used a virus vector which expressed destabilized GFP under the E-SARE promoter, and cortical layer 2/3 pyramidal neurons in the barrel cortex were recorded (Kawashima et al., 2013). When the whisker-evoked activity and spontaneous activity were measured, interestingly, only the whisker-evoked activity showed a significant correlation with reporter GFP expression; in sharp contrast, spontaneous activity revealed little correlation (**Figure 2**). This labeling selectivity for neuronal activity constituted a characteristic feature of E-SARE activation (Kawashima et al., 2013). These results highlighted the critical importance of choosing the right type of the activity-dependent promoters and reporter proteins to correctly target the neural activity to be studied.

Multiple lines of evidences suggest that neurons that were marked with activity-dependent promoters might undergo robust changes in electrophysiological and anatomical properties when animals are exposed to various memory-related tasks. In one study, FosGFP mice were trained with repetitive “trace” fear conditioning, and an increase in postsynaptic calcium-permeable AMPA receptors (CP-AMPAR) was exclusively observed in FosGFP-positive neurons, but not in FosGFP-negative neurons, in the anterior cingulate cortex (Descalzi et al., 2012). When FosGFP mice were exposed to repetitive administration of cocaine to evoke psychomotor sensitization, a sign of cocaine addiction, “silent synapses” containing functional NMDA receptors but few functional AMPA receptors were significantly increased in FosGFP-positive neurons in the nucleus accumbens (Koya et al., 2012). Furthermore, a third study showed that upon establishment of contextual fear conditioning, GFP-GluR1^*c-fos*^ transgenic mice, which express GFP-GluR1 downstream of tetracycline transactivator (tTA) driven by c-*fos* promoter (Matsuo et al., 2008), revealed a selective decrease of dendritic spines in GFP-positive neurons in the CA1 neurons of the hippocampus (Sanders et al., 2012). These studies, together, demonstrate the usefulness of activity-dependent promoters in identifying a small number of neurons in which important electrophysiological and morphological changes do occur, which otherwise would have been inevitably missed. Furthermore, this illustrates the reliability of activity-dependent promoters in specifically labeling behaviorally relevant neurons among a majority of functionally heterogenous neurons.

## BEHAVIORAL MANIPULATION OF COGNITION IN LIVING ANIMALS BASED ON ACTIVITY-DEPENDENT PROMOTERS

Following recent advancement of genetic and chemical methods for manipulation of neural activity, activity-dependent promoters are now widely being used to modulate neural circuits which underlie cognitive behaviors in living animals. In a pioneering study, Reijmers et al. (2007) developed a “TetTag” method based on a tTA (Gossen and Bujard, 1992). This combinatorial strategy takes advantage of a first transgenic mouse line in which tTA was expressed downstream of c-*fos* promoter, and a second transgenic mouse line in which a reporter LacZ protein was expressed downstream of tetracycline responsive element (TRE) which is activated by tTA. This double-transgenic design enabled prolonged marker expression (<1–2 weeks) in neurons which were activated during a specific time period that was experimentally defined by doxycycline administration. Based on this system, they found that a subset of the basolateral amygdala (BLA) neurons was activated at the acquisition of fear memory and, 3 days later, the same population was again activated during the retrieval of memory (Reijmers et al., 2007).

This TetTag method has paved the way for a new series of behavioral studies based on manipulation of functionally defined neurons in behaving animals. These studies involve two steps of experiments. First, a specific neuronal subset is labeled using TetTag under a certain behavioral condition through combination of an activity-dependent promoter and a time-restrictive drug administration. The resulting reporter proteins, which persist for several days, are subsequently used for controlling the activity of labeled neurons and their effects on behaviors are examined. A couple of recent studies (Liu et al., 2012; Ramirez et al., 2013) adopted the TetTag system in combination with with AAVs expressing light-gated cation channel channelrhodopsin-2 (ChR2; Boyden et al., 2005), to specifically label activated hippocampal dentate gyrus neurons during the acquisition of contextual fear memory. When this ChR2-TetTag system was combined with aversive experiences (foot shocks), light-driven excitation of the labeled neurons evoked an “artificial” fear response, demonstrating that the reactivation of an “engram” of fear memory was sufficient for memory recall in the hippocampus (Liu et al., 2012). Another study (Garner et al., 2012) used a mouse which expressed designer receptors exclusively activated by designer drugs (DREADD), hM3Dq (Alexander et al., 2009), downstream of TRE. Neurons that were activated in one context was first labeled with hM3Dq in the absence of chemical stimuli, and when these neurons were chemically excited during acquisition of fear memory in another context, a “synthetic memory” was formed in association with the first context (Garner et al., 2012).

An original chemical genetic approach was used by Koya et al. (2009). In this study, a chemical compound Daun02, which is converted into a neuronal silencer daunorubicin by beta-galactosidase activity of LacZ, was used in combination with Fos-LacZ rats. Local injection of Daun02 incapacitated a dominant subset of nucleus accumbens neurons which were previously activated by cocaine administration and thus impaired the formation of context-specific psychomotor sensitization, indicating the presence a functional sub-network which associates the environment and addiction behaviors (Koya et al., 2009).

These newly discovered aspects of learned behaviors demonstrate the unique power of functional labeling based on activity-dependent promoters over conventional anatomy- or cell-type-based labeling. The majority of the publications that used functional labeling so far employed this technique to mark a hippocampal circuit that participated in encoding of external contexts during fear conditioning. However, we recently showed that the scope of functional labeling can be extended to different types of circuits, such as stimulus feature (orientation)-specific circuits in the visual cortex (Kawashima et al., 2013). We anticipate that many exciting discoveries lie ahead when it will become common to selectively manipulate functional neuronal ensembles that are associated with various brain functions.

## PERSISTENT LABELING OF FUNCTIONAL NEURONS USING ACTIVITY-DEPENDENT PROMOTERS

One new direction of functional labeling involves conversion of transient expression from activity-dependent promoters into a permanent labeling based on tamoxifen-dependent recombinases, such as CreER^T2^ (Feil et al., 1997) or ER^T2^-Cre-ER^T2^ (Matsuda and Cepko, 2007). This technique uses two transgene cassettes: in the first cassette, a tamoxifen-dependent Cre recombinase is expressed under control of an activity-dependent promoter, and in the second cassette, a “STOP signal”, which is flanked by recombination sequences [such as single loxP or double loxP (such as DIO or FLEX)], is placed between the reporter gene of interest and a constitutive promoter. Upon tamoxifen administration within a sharp time window when the drug-sensitive Cre recombinases are expressed in activated neurons, these recombinases excise the “STOP” signal from the second cassette and the reporters will then be expressed constitutively. One group developed several knock-in mice, called targeted recombination in active population (TRAP), which express CreER^T2^ in the endogenous c-*fos* and *Arc* locus (Guenthner et al., 2013). Independently, we developed a combinatorial AAV-based system which expresses ER^T2^-Cre-ER^T2^ downstream of the synthetic E-SARE promoter (Kawashima et al., 2013).

Chronic labeling of once-activated neuronal ensembles using activity-dependent promoters holds huge promises in neuroscience. For example, persistent expression of fluorescent tracers which was induced by E-SARE-driven ER^T2^-Cre-ER^T2^ successfully labeled and allowed live imaging of long-distance (>3 mm) axons projecting from eye-specific neurons in the lateral geniculate nucleus (LGN) to the layer 4 of the visual cortex (Kawashima et al., 2013). This validates the usefulness of activity-dependent promoters even in functional connectomics studies, where long-distance axons from mixed, but functionally segregated neurons, could be traced to multiple independent target regions. We anticipate that these new toolkits for chronic labeling of active ensembles will provide a much awaited experimental basis to interrogate various aspects of neuronal circuits underlying long-term plastic changes of the brain, such as during nervous system development, during establishment of long-lasting remote memory over months, or in association with age-related neuronal changes over several years.

## FUTURE PERSPECTIVES

While vastly improved over the past, the current repertoire of activity-dependent promoters can still be significantly improved. For example, controlling the activity-dependent promoters through drug-controlled transcriptional silencers (Freundlieb et al., 1999) or light-controlled transcription repressors/activators (Wang et al., 2012; Konermann et al., 2013) will enable a much more precise temporal restriction of the neuronal activity that triggers activity-driven expression of the reporter protein. Also, further enhancement of expression levels or dynamic ranges of activity-regulated promoters might be possible through large-scale unbiased mutational screenings (Melnikov et al., 2012). As the developments of a comprehensive genetic toolkit based on activity-dependent promoters progresses, the range of applications for imaging, manipulating and chronically labeling an active ensemble will undoubtedly continue to expand at a fast pace, while also creating massive opportunities to shed new lights on complex animal behaviors that are driven by mixed ensembles of active neurons in heterologous neuronal circuits in multiple brain areas.

## Conflict of Interest Statement

The authors declare that the research was conducted in the absence of any commercial or financial relationships that could be construed as a potential conflict of interest.
